# Evaluation of Chitosan Hydrogel for Sustained Delivery of VEGF for Odontogenic Differentiation of Dental Pulp Stem Cells

**DOI:** 10.1155/2019/1515040

**Published:** 2019-12-19

**Authors:** Si Wu, Yachuan Zhou, Yi Yu, Xin Zhou, Wei Du, Mian Wan, Yi Fan, Xuedong Zhou, Xin Xu, Liwei Zheng

**Affiliations:** ^1^State Key Laboratory of Oral Diseases, National Clinical Research Center for Oral Diseases, Department of Pediatric Dentistry, West China Hospital of Stomatology, Sichuan University, Chengdu, Sichuan 610041, China; ^2^State Key Laboratory of Oral Diseases, National Clinical Research Center for Oral Diseases, Department of Cariology and Endodontics, West China Hospital of Stomatology, Sichuan University, Chengdu, Sichuan 610041, China

## Abstract

The pulpotomy with pulp capping is aimed at retaining vital pulp with reparative dentin formation. Vascular endothelial growth factor (VEGF) plays a crucial role in dentin regeneration; however, its constant administrations in the human body is still problematic. Chitosan was widely studied as an effective carrier to deliver bioactive molecules in regenerative medicine. In this study, we conducted a chitosan/*β*-glycerophosphate (CS/*β*-GP) hydrogel as a VEGF-sustained release system and explored its effects on dental pulp stem cells (DPSCs). CS/*β*-GP hydrogel was manufactured using a sol-gel method. SEM assay showed the spongy and porous microstructure of the lyophilized hydrogels. DPSCs cultured in the CS/*β*-GP hydrogel kept adhesion and vitality. CCK-8 assay tested the promoted proliferation activity of DPSCs on the hydrogel. Besides, the added VEGF protein could continually release from VEGF/CS/*β*-GP hydrogel. The VEGF/CS/*β*-GP hydrogel could promote the odontogenic differentiation of DPSCs better than VEGF treatment without hydrogel. Our results suggested that CS/*β*-GP hydrogel could continually release VEGF and contribute to odontogenic differentiation of DPSCs, thus may become a potential carrier of bioactive molecules in pulp capping therapy.

## 1. Introduction

The dental pulpotomy is a kind of dental therapy to retain the vital pulp in accidental pulp exposure caused by trauma or caries removal. The retained radical pulp is valuable for continuous apexogenesis in young permanent teeth with immature root. In pulpotomy, the infected coronal pulp is amputated, and the surface of remaining vital pulp is treated with a sealant, such as calcium hydroxide or mineral trioxide aggregate (MTA) [1]. These sealants, called pulp capping agents, can promote the recruitment, migration, proliferation, and differentiation of human dental pulp stem cells (DPSCs) [2]. Afterwards, a protective mechanism is initiated. The dentin matrix secreted by odontoblast-like cells is laid down on the surface of amputated pulp. As a result, the dentin bridge or osteodentin is formed to save the vitality of residual pulp [2].

However, as widely used capping agents, the calcium hydroxide has been evaluated with less success in long-term studies, while MTA has drawbacks such as discolor of tooth, high cost, high operational requirements, and longer curing time [3]. Considering the mechanism underlying the reparative dentin formation, bioactive molecules were studied to promote the proliferation and differentiation potential of DPSCs in vital pulp tissue [4–10].

Vascular endothelial growth factor (VEGF) plays a crucial role in dentin formation and regeneration [11]. Studies have evaluated that VEGF can promote the odontogenic differentiation of cultured DPSCs and induce the formation of reparative dentin on the surface of amputated pulp [12–16]. However, the applicable VEGF recombinant protein has a short half-life in aqueous solutions at 37°C [17]. Most recombinant proteins are susceptible to high temperature or pH levels, and they will be easily degraded by enzymes and loss efficiency. Nowadays, some growth factors have been approved for human therapy as recombinant preparations; however, most of them still carry warnings on clinical application. The use of recombinant proteins without any carriers generally presents side effects to human body. These proteins are pleiotropic with short half-lives and sometimes functional redundancies and overlapping side effects [18–20]. Many researches and therapies require frequent protein administration and ultimately poor patient compliance [21]. The systemic application of proteins with once large dose or frequent administration may induce a range of flu-like symptoms as well as more severe hematologic, autoimmune, infection, and dermatologic adverse events [18, 22].

In order to effectively extend the residence time and optimize the molecule's concentration, various materials were studied as carriers to deliver bioactive molecules in pulp capping therapy [6, 23, 24]. The carriers have different features like synthetic gel (hydrogel), sponges, scaffolds, and membranes [7, 8, 25–27]. Only the sustained delivery carrier can create a microenvironment to maintain a certain molecule concentration and extend application period. In other words, the carriers could prolong the effective period and minimize the side effects [4, 28].

Chitosan is a kind of polysaccharides derived from chitin which is a natural component of insects' exoskeleton, crustaceous shells, and fungi's cell walls. Chitosan has characteristics of bacteriostatic effects, nontoxicity, and biocompatibility [23]. In pharmaceutical industry, chitosan has been widely used as a drug delivery system in different forms, like tablets, microspheres, hydrogels, and nanoparticles [20]. Among these, the chitosan/*β*-glycerophosphate (CS/*β*-GP) hydrogel gained attention by its excellent chemical and biological ability to deliver therapeutic agents, molecules, or cells. It has been studied in cartilage repair, bone regeneration, hemostatic agents, and even in endodontic treatment [19, 29–32]. In the study of odontology, chitosan shows good properties as a carrier for some medicaments, such as chlorhexidine, calcium hydroxide, and triple antibiotic paste [33]. The temperature-sensitive CS/*β*-GP solution can transform into semisolid hydrogel at physiological temperature in human bodies. Besides, the hydrogel protects the agents from physiological degradation and prolongs therapeutic span while minimizing side effects [20].

In this study, we characterized the morphology of CS/*β*-GP thermosensitive hydrogel and the bioactivity of dental pulp stem cells (DPSCs) on the hydrogel. We also compared the effects of VEGF treatment in CS/*β*-GP hydrogel and without hydrogel on the behaviors of DPSCs. We hypothesized that the thermosensitive chitosan hydrogel could effectively deliver VEGF protein in a sustained release pattern to stimulate differentiation and mineralization of DPSCs.

## 2. Materials and Methods

### 2.1. Isolation and Culture of Dental Pulp Stem Cells

The procedures were approved by the Ethical Committee of the West China School of Stomatology, Sichuan University, and performed in accordance with the approved guidelines. Human dental pulp stem cells (DPSCs) were harvested from normal impacted third molars extracted from donors (19–22 years old) in West China Hospital of Stomatology and cultured as previously described [34]. All donors provided informed consent for this study. DPSCs were cultured in Dulbecco's modified Eagle's medium (DMEM) consisting of 10% fetal bovine serum (FBS) and 1% penicillin/streptomycin (PS) at 37°C in moist atmosphere with 5% CO_2_ for use. Cells between passages 3 and 4 were used in this study.

To characterize the immunophenotype of DPSCs, flow cytometric analysis was used to measure the expression of mesenchymal and nonmesenchymal stem cell-associated surface markers at passages 3. DPSCs were washed by PBS and liberated by enzymatic digestion for 2 minutes at 37°C. Then, the single cell suspension was washed twice by buffer solution (PBS containing 5% BSA). DPSCs for immunolabeling were resuspended in 0.5 ml blocking buffer and incubated on ice for 30 minutes. Tubes containing 1 × 10^6^ of DPSCs were incubated with appropriate antibodies (CD90: 328109, CD29: 303003, CD45: 368507, and CD34: 343603, BioLegend) away from light on ice. The control group was incubated without antibodies in buffer solution. After 30 minutes, cells were washed twice by buffer solution and analyzed on Cytomics™ FC 500 (Beckman Coulter Ltd.).

### 2.2. Fabrication of Hydrogel and VEGF Loading

Chitosan (CS, viscosity: 200-400 mPa·s) was obtained from Aladdin Industrial Corporation (China). Acetic acid and *β*-glycerophosphate (*β*-GP) were purchased from Sigma (St. Louis, USA). The 2% (*w*/*v*) chitosan solution was prepared by stirring chitosan in 0.5% (*v*/*v*) acetic acid solution at room temperature for at least 3 hours until complete dissolution. Afterwards, the chitosan solution was stored overnight at 4°C to diminish inside bubbles. 56% (*w*/*v*) beta-sodium glycerophosphate (*β*-GP) solution was prepared by mixing *β*-GP with distilled water and then filter sterilized by a 0.22 diameter filter. These two solutions were mixed by adding the *β*-GP drop by drop into the stirring chitosan solution; the volume ratio of CS: *β*-GP is 5/1 [31]. After magnetic stirring for 10 minutes under ice bath, the final pH value of the chitosan solution was 7.49. After that, the VEGF/CS/*β*-GP hydrogel was obtained by adding appropriate amount of recombinant human VEGF protein (PeproTech, China) into CS/*β*-GP solution under magnetic stirring for 10 minutes; the final concentration of VEGF was 100 ng/ml.

During gelation, these gel solutions were transferred to 37°C baths for 10 minutes. The process of sol-gel transition was observed.

### 2.3. Scanning Electron Microscope (SEM) of the Hydrogel and DPSCs

After gelation in glass containers, hydrogels were lyophilized. The samples were cut into pieces, and the microstructures were observed by SEM (JEOLJEM-1400, Japan) at an acceleration voltage of 20.00 kV. DPSCs were directly seeded and cultured on the surface of CS/*β*-GP hydrogels. After 24 hours, cell-seeded gels were washed with phosphate buffered saline (PBS) for 3 times and fixed with 2.5% glutaraldehyde at room temperature for 4 hours. Then, the hydrogels were dehydrated in a graded series of ethanol (30%, 50%, 75%, 85%, 95%, and 100%) for 15 minutes in each concentration and air-dried overnight to be analyzed by SEM (JEOLJEM-1400, Japan).

### 2.4. Cell Viability Using AO/EB Staining

CS/*β*-GP gel solution was put in the 6-well culture plates for 1 ml/well. After gelation, the culture medium was added into the wells to soak the hydrogels for 10 minutes for 3 times. Then, DPSCs were suspended and cultured on the surface of hydrogels at a density of 10^6^ cell/well. After 24-hour culture, cells on the surface of hydrogels were stained by 1 *μ*l/0.1 ml AO/EB (acridine orange/ethidium bromide) solution (Sabbiotech) for 1 minute. The images were captured on a Nikon Eclipse 300 fluorescence microscope (Compix Inc.).

### 2.5. Cytotoxicity Using Cell Counting Kit-8 Assay

The cytotoxicity of CS/*β*-GP hydrogel was assessed using a Cell Counting Kit-8 (CCK-8, Sigma, St. Louis, MO, USA). DPSCs were cultured in hydrogel leachates or seeded on the surface of hydrogels. The leachates of hydrogels were obtained using an international standard procedure (ISO-10993) [29]. DPSCs were seeded in 96-well culture plates at a density of 2000 cells/well. The medium was replaced by a fresh culture medium or hydrogel leachates every 24 hours. After 1, 3, 5, and 7 days, cells were isolated and incubated with 10 *μ*l/0.1 ml CCK-8 solution and then tested using a BioTek ELX800 kit (BioTek, Winooski, VT, USA) in an absorbance of 450 nm.

### 2.6. Release Behaviors of VEGF

The VEGF/CS/*β*-GP hydrogel leachates were obtained using an international standard procedure (ISO-10993). The leaching solution was collected and immediately frozen at -80°C. The same volume of PBS was replenished. The concentrations of VEGF in the leaching solution were measured by using the enzyme-linked immunosorbent assay (ELISA) kit (Dakewe Biotech Company Limited, China). The optical densities were measured at 450 nm using BioTek ELX800. The standard curves were plotted, and the concentrations of VEGF were calculated compared with the standard curves and stated in ng/ml.

### 2.7. ALP and Alizarin Red Staining

DPSCs were cultured in 24-well plates and treated with four different concentrations of VEGF protein (5, 10, 50, and 100 ng/ml) in odontogenic medium (OM, consisting of DMEM, 10% FBS, 1%PS, 10 mmol l^−1^*β*-GP, 50 *μ*g/ml ascorbic acid 2-phosphate, and 10^−7^ mol/l dexamethasone). DPSCs in base culture medium (NC, consisting of DMEM, 10% FBS, and 1% PS) were cultured as a negative control group. DPSCs in OM without VEGF were as another control group. Cells were dyed using an alkaline phosphatase (ALP) staining kit (Beyotime, China) after 0, 4, and 7 days, and alizarin red staining (ARS) after 7 and 14 days. For quantitative analysis, 10% (*w*/*v*) cetylpyridinium chloride resolution was used to elute the alizarin red positive depositions. The absorbance was measured using BioTek ELX800 (BioTek, Winooski, VT, USA) in an optical density of 562 nm.

The VEGF/CS/*β*-GP hydrogels were placed on the upper chambers, and DPSCs were cultured on the lower chambers in transwell plates. In the 100 ng/mL VEGF group, DPSCs were cultured in OM containing same amount of VEGF (100 ng/ml) without hydrogels for seven days. DPSCs in NC and OM groups were cultured without hydrogels. DPSCs were dyed using the ALP staining kit after 4 and 7 days, and ARS after 10 and 14 days. Before staining, cells were washed by PBS for 3 times and fixed in 4% paraformaldehyde for 15 minutes in room temperature. The stained cells were observed under light microscopy.

### 2.8. RNA Extraction and qRT-PCR

Total RNAs of DPSCs were extracted using TRIzol reagent according to the manufacturer's protocol. Reverse transcription was performed with a PrimeScript® RT reagent kit with gDNA Eraser (TaKaRa). Quantitative real-time PCR (qRT-PCR) was carried out using a standard SYBR Green PCR kit (TaKaRa) on a CFX96 (Bio-Rad). Glyceraldehyde-3-phosphate dehydrogenase (GAPDH) was used to normalize the expression level of each gene. The primer information is shown in Table 1.

### 2.9. Western Blot Analyses

Total proteins of DPSCs were extracted following the kit (KeyGEN, China) protocol. After protein denaturalization, the protein concentrations were measured by bicinchoninic acid (BCA) protein assays (Beyotime, China). Equal amount of each sample was segregated via sodium dodecyl sulfate polyacrylamide gel electrophoresis (SDS-PAGE) gels and then transferred to a nitrocellulose membrane. After blocking, the membranes were incubated with primary antibody: mouse anti-*β*-actin (ab8226, Abcam, 1 : 1000) and mouse anti-OSX (sc-393325, Santa Cruz Biotechnology, 1 : 1000). Then, the membranes were incubated with goat anti-mouse IgG-horseradish peroxidase (Santa Cruz Biotechnology) and detected with a chemiluminescent reagent kit (Millipore). The expression level of *β*-actin was normalized. A GS-700 imaging densitometer (Bio-Rad) was used for image analysis.

### 2.10. Statistical Analysis

The results are revealed as mean ± SD from experiments conducted at least 3 times independently and analyzed by two-way ANOVA with SPSS 21.0. When the *P* values were <0.05, data were considered statistically significant. ^∗^*P* < 0.05, ^∗∗^*P* < 0.01, and ^∗∗∗^*P* < 0.005.

## 3. Results

### 3.1. Gelation and Microstructure of Hydrogels

**Figure 1:**
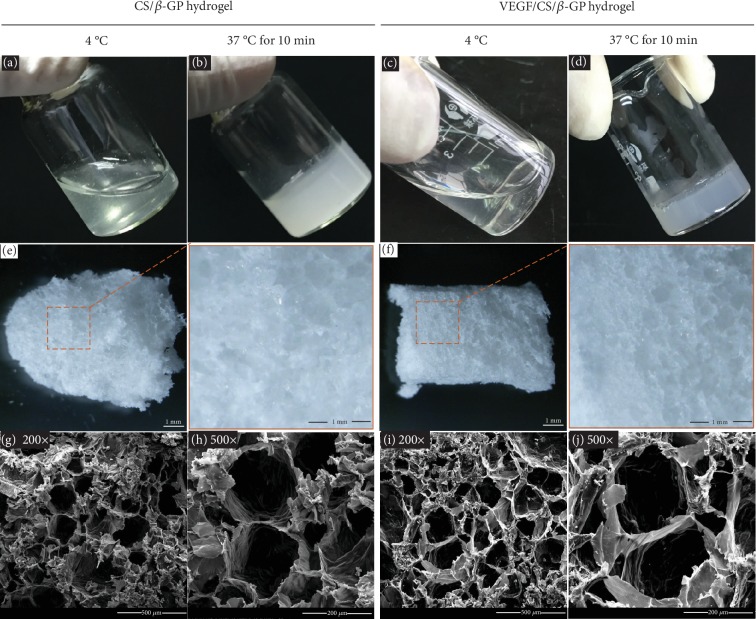
The process of gelation and the microstructure of hydrogels. Photographs of CS/*β*-GP gel before and after gelation (a, b). Photographs of VEGF/CS/*β*-GP gel before and after gelation (c, d). Photographs of CS/*β*-GP and VEGF/CS/*β*-GP hydrogels after lyophilization (e, f). SEM images of CS/*β*-GP hydrogel in 200x and 500x (g, h). SEM images of VEGF/CS/*β*-GP hydrogel in 200x and 500x (i, j).

The CS/*β*-GP gel solution was prepared as procedures described previously [22]. The VEGF/CS/*β*-GP gel solution was formed by adding VEGF protein into CS/*β*-GP solutions. The gel solution was transparent liquid at 4°C and transformed into nontransparent semisolid hydrogel after incubation at 37°C for 15 minutes (Figures 1(a)–1(d)). After gelation, the CS/*β*-GP and VEGF/CS/*β*-GP hydrogels were lyophilized and observed by SEM (Figures 1(e) and 1(f)). These lyophilized hydrogels showed the spongy and porous microstructure and the average pore diameter range from 100 to 200 *μ*m (Figures 1(g)–1(j)). There was no significantly different appearance of hydrogels with or without VEGF proteins.

### 3.2. Adhesion of DPSCs on the Hydrogel

**Figure 2:**
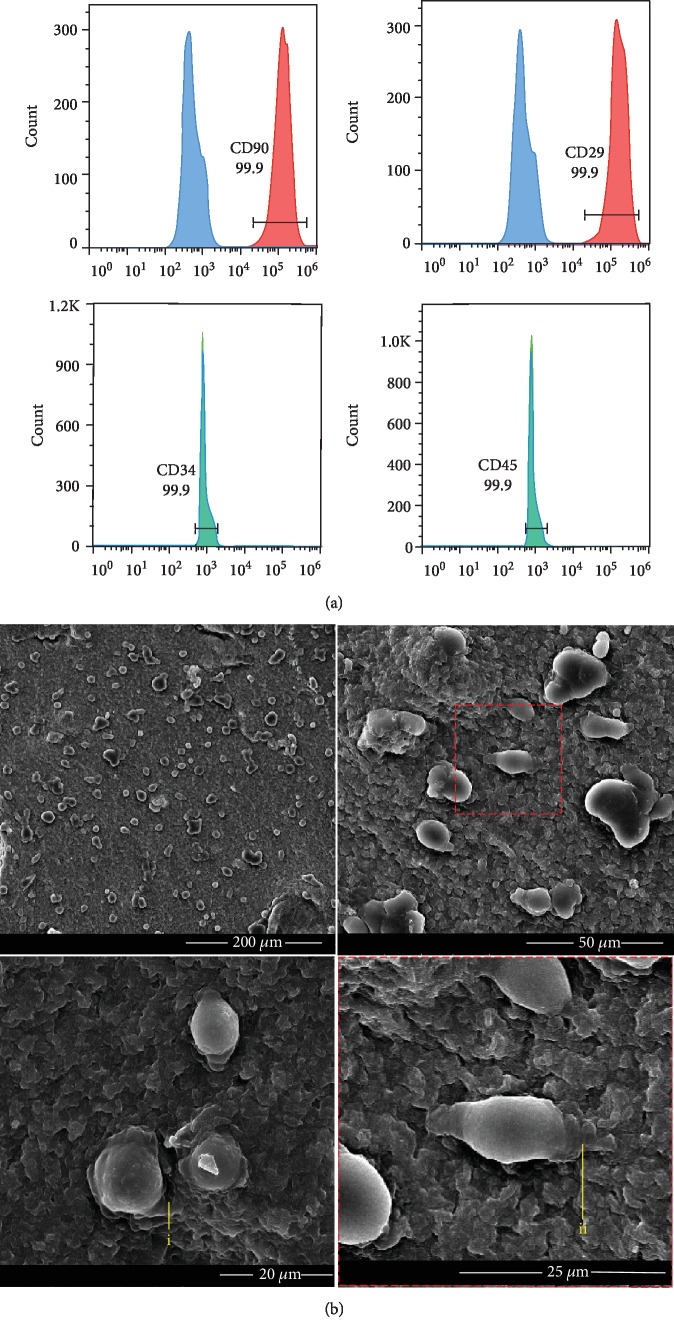
Cell surface markers on DPSCs and the morphology of DPSCs cultured on the hydrogel. Flow cytometric analysis was used to test the surface markers of DPSCs. DPSCs were positive for CD29 and CD90, and negative for CD34 and CD45 (a). Morphology of DPSCs cultured on the surface of CS/*β*-GP hydrogel after 24 h (b). DPSCs embedded their cellular synapses into the pore canal (i, ii).

The flow cytometry detected that the cultured DPSCs were positive for CD29 and CD90, and negative for CD45 and CD34, which are the criteria for mesenchymal stem cell (Figure 2(a)). The DPSCs were planted on the surface of CS/*β*-GP hydrogel for 24 hours. The microstructure of CS/*β*-GP hydrogel with DPSCs was analyzed by SEM. DPSCs showed spherical shapes, and the cellular synapses were embedded into the porous hydrogel (Figure 2(b), i and ii).

### 3.3. Cytotoxicity of CS/*β*-GP Hydrogel to DPSCs

**Figure 3:**
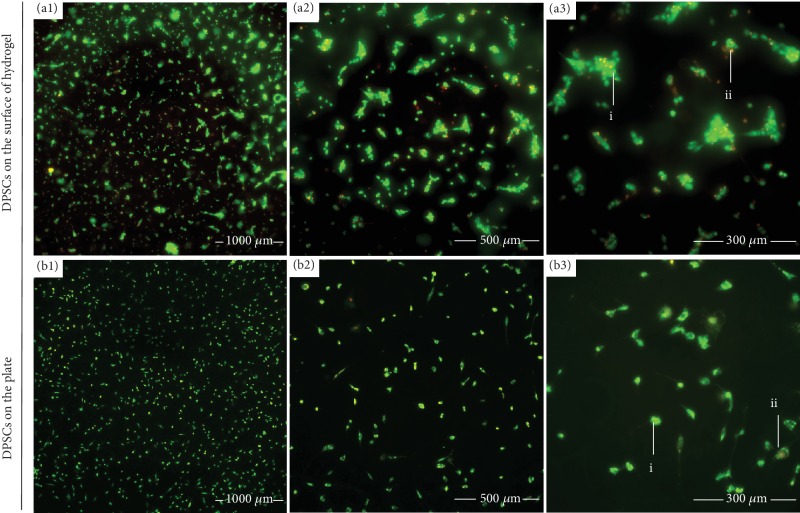
The activity of DPSCs cultured on the hydrogel. Distribution and viability of DPSCs cultured on the surface of CS/*β*-GP hydrogel or on well plates after 24 hours stained by AO/EB. Live cells were shown in green or yellow-green (i), and apoptotic cells were red or orange (ii).

AO/EB double fluorescence staining was conducted to observe the morphology, distribution, and viability of DPSCs cultured on the surface of CS/*β*-GP hydrogel after 24 hours (Figure 3(a)). DPSCs cultured without hydrogel were as control groups (Figure 3(b)). Live cells were stained in green or yellow-green (Figure 3 (i)), and apoptotic cells were red or orange (Figure 3 (ii)). There was no significant difference of cell population in the CS/*β*-GP hydrogel group compared to cells without hydrogel, and most of DPSCs on the hydrogel kept vitality.

**Figure 4:**
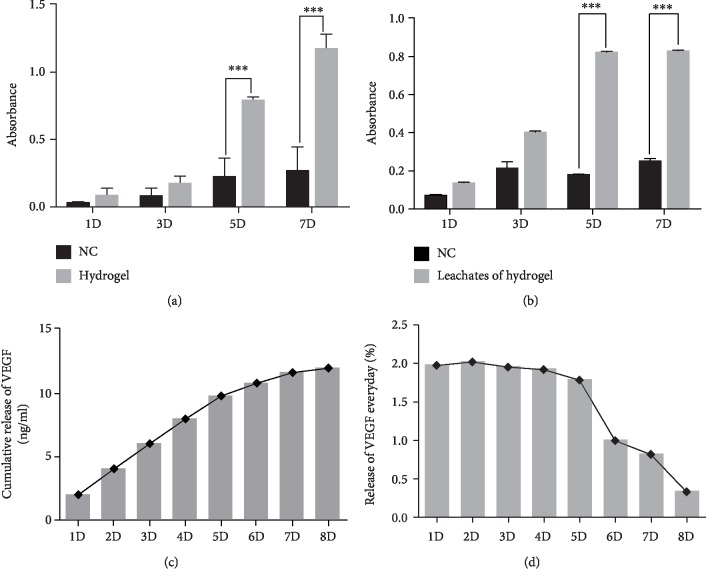
The CS/*β*-GP hydrogel promoted the proliferation of DPSCs and constantly released VEGF. The results of CCK-8 assay showed the promoted proliferation of DPSCs plated on the CS/*β*-GP hydrogel and in hydrogel leachates (a, b). DPSCs cultured in base culture medium (NC) without hydrogels or hydrogel leachates were as controls. ELISA assay showed the cumulative release profiles of VEGF/CS/*β*-GP hydrogel (c). ELISA assay showed the release amount of VEGF from hydrogel every day (d).

Cell Counting Kit-8 (CCK-8) assay was conducted to test the cytotoxicity of CS/*β*-GP hydrogel. The proliferation of DPSCs cultured on the surface of hydrogel (Figure 4(a)) and in the hydrogel leachates (Figure 4(b)) was assayed. Cells cultured on the plate were as control groups. The activity of DPSCs showed no difference on the 1^st^ and 3^rd^ day. Surprisingly, the promoted proliferation of DPSCs was shown in hydrogel and hydrogel leachate groups compared to the control group after 7 days. These results suggested that the CS/*β*-GP hydrogel was noncytotoxic; furthermore, it has the characteristic to promote the proliferation of DPSCs.

### 3.4. VEGF Release from CS/*β*-GP Hydrogel

VEGF proteins were added into the CS/*β*-GP hydrogel to form 100 ng/ml VEGF/CS/*β*-GP hydrogel, and the release profiles of VEGF were detected using the enzyme-linked immunosorbent assay (ELISA). As a result, a linear increase of VEGF release was observed during the first 5 days. After 8 days, the cumulative release level tended to the peak and levelled out. A total of 12% VEGF proteins were shown to release out of hydrogel after 8 days (Figure 4(c)). The everyday release of VEGF proteins showed a downward trend from the 4th day to reach a constant concentration (Figure 4(d)). The results suggested that the CS/*β*-GP hydrogel could be used as a carrier to constantly release VEGF proteins.

### 3.5. The Sustained Release of VEGF from Hydrogels Promoted the Odontogenic Differentiation of DPSCs

**Figure 5:**
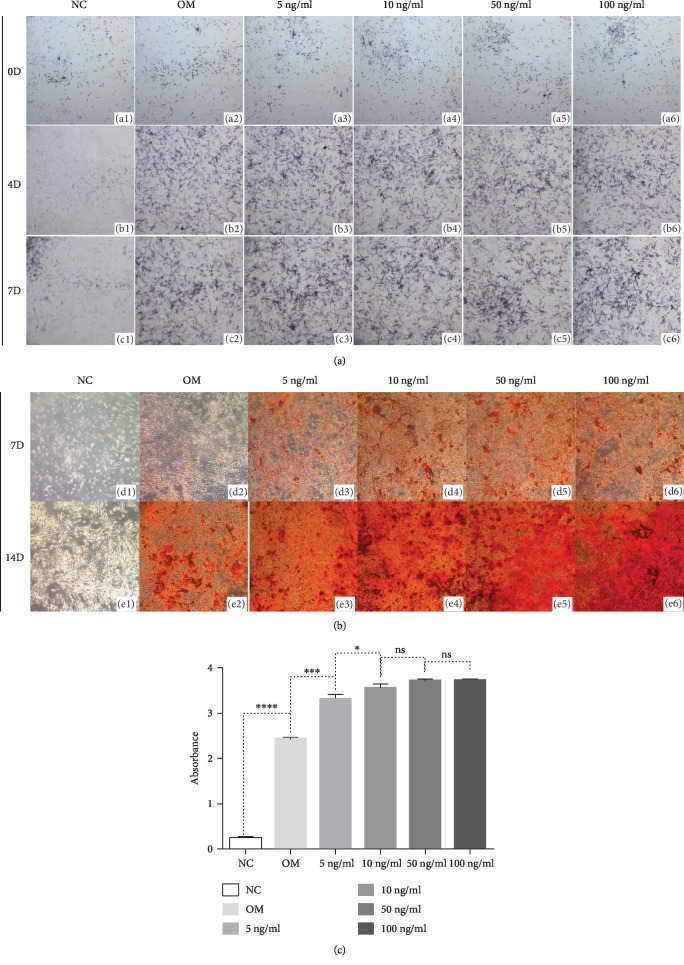
VEGF promoted odontogenic differentiation in DPSCs. The induced ALPase activity was tested in DPSCs treated with VEGF compared to cells without VEGF (a). VEGF treatment at 10 ng/ml concentration produced more mineralized nodules than 5 ng/ml VEGF treatment, and cells with 10 ng/ml, 50 ng/ml, and 100 ng/ml did not show an obvious difference in the amount of mineralized nodules (b). The quantitative analysis of ARS was conducted (c). NC: base culture medium; OM: odontogenic culture medium.

VEGF could promote odontogenic differentiation of DPSCs, while the strategy of optimal concentration treatment remains unclear. The effects of VEGF treatment in DPSCs were detected using different concentrations of 5 ng/ml, 10 ng/ml, 50 ng/ml, and 100 ng/ml. The results of ALP staining illustrated induced ALPase activity in DPSCs treated with VEGF compared to cells without VEGF (Figure 5(a)). After 7 days, the VEGF treatment significantly increased the mineralized nodule formation (Figure 5(b)). Cells cultured with 10 ng/ml VEGF exhibited to be higher mineralized than cells with 5 ng/ml VEGF, and cells with 10 ng/ml, 50 ng/ml, and 100 ng/ml did not show an obvious difference in the amounts of mineralized nodules (Figure 5(c)). It suggested that more than 10 ng/ml VEGF may not be needed to induce the odontogenic differentiation of DPSCs, and this result was consistent with previous study [14].

**Figure 6:**
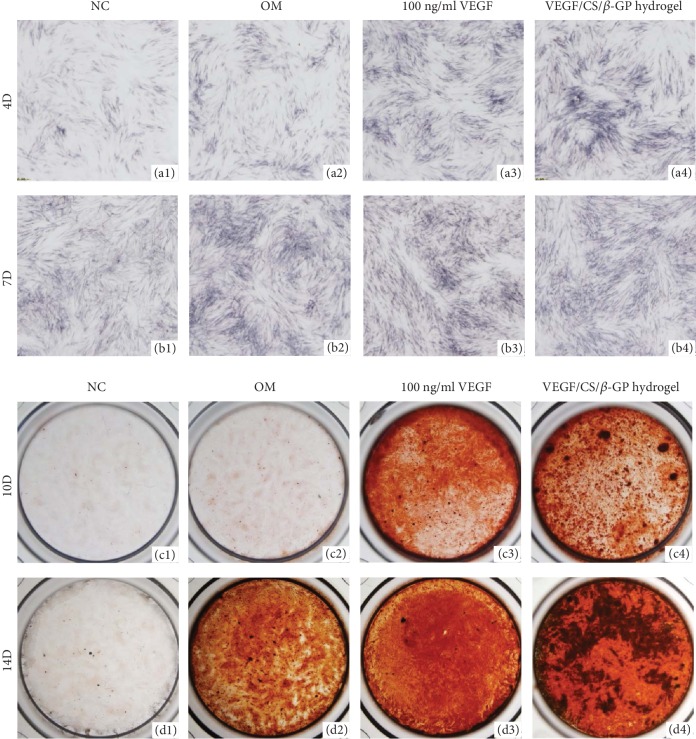
The sustained release of VEGF from hydrogels promoted the odontogenic differentiation of DPSCs. The results of ALP staining showed that the addition of VEGF protein in the medium and in the hydrogel both increased ALPase activities of DPSCs after 7 days (a1–a4, b1–b4). The sustained release of VEGF in VEGF/CS/*β*-GP hydrogels elevated the formation of mineralized nodules better than the initial burst release of VEGF in the medium (c1–c4, d1–d4). NC: base culture medium; OM: odontogenic culture medium.

The CS/*β*-GP hydrogel was evaluated as a valuable sustained delivery system for bioactive molecule release. To further investigate the advantage of hydrogel system compared to the once-add strategy without carriers, we evaluated the cell responses to 100 ng/ml VEGF proteins with or without a CS/*β*-GP delivery system. Cells without hydrogel cultured in NC and OM were as controls. The results of ALP staining showed that the addition of VEGF protein in the medium and in the hydrogel both increased ALPase activities after 7 days, and no obvious difference was shown between two groups (Figures 6(a1)–6(a4) and 6(b1)–6(b4)).

ARS was further performed to detect the mineralization activity of DPSCs during the late stage of differentiation. After 10 days, the added VEGF proteins increased the formation of mineralized nodules compared to control groups (Figures 6(c1)–6(c4)). Moreover, the sustained VEGF treatment elevated the mineralization activity of DPSCs better than the initial burst release of VEGF without carriers (Figures 6(d3) and 6(d4)). The hydrogel worked as a sustained delivery system and created a steady concentration of VEGF protein, promoting the odontogenic differentiation of DPSCs in the long-term differentiation period.

**Figure 7:**
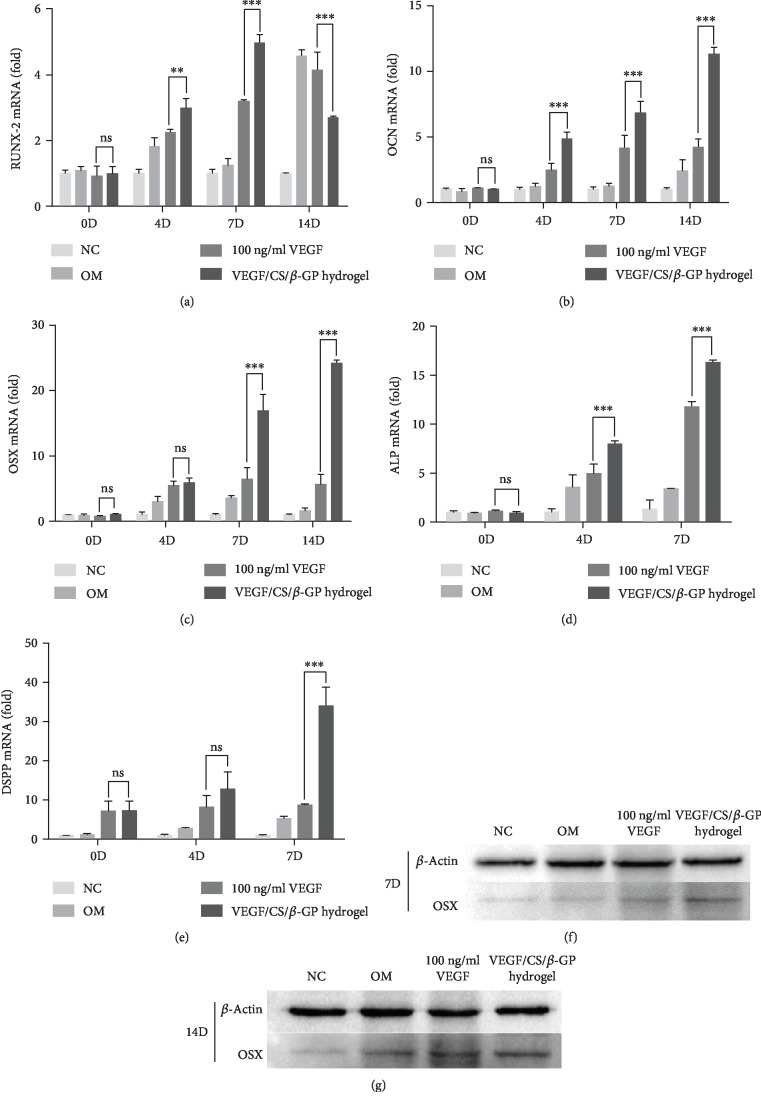
The sustained release of VEGF from hydrogels elevated odontogenic marker expressions in DPSCs. The expression levels of RUNX-2, OCN, and OSX during the odontogenic differentiation of DPSCs (a–c). The ALP expression level during the early stage of differentiation in DPSCs (d). The DSPP expression level during the late stage of differentiation in DPSCs (e). Data are presented as mean ± SD. ns: no significant, ^∗∗^*P* < 0.01, ^∗∗∗^*P* < 0.005. The expression levels of OSX protein in DPSCs after 7 and 14 days (f, g). NC: base culture medium; OM: odontogenic culture medium.

The expressions of odontogenic markers were further detected using qRT-PCR assay. The alkaline phosphatase (*ALP*) expression level was higher in the VEGF/CS/*β*-GP hydrogel group than other groups, in consistent with the results of ALPase staining (Figure 7(d)). The expression levels of osteocalcin (*OCN*), osterix (*OSX*), and dentin sialophosphoprotein (*DSPP*) were significantly higher in the VEGF/CS/*β*-GP hydrogel group after 7 days compared to the 100 ng/ml VEGF group (Figures 7(b), 7(c), and 7(e)). The expression of runt-related transcription factor-2 (*RUNX-2*) increased at the 7^th^ day while decreased at the 14^th^ day in the VEGF/CS/*β*-GP group (Figure 7(a)). It validated the VEGF/CS/*β*-GP hydrogel delivery system induced the odontogenic differentiation of DPSCs.

Consistent with the results in gene expression, the protein expressions of osterix (*OSX*) were also increased in the DPSCs cocultured with VEGF/CS/*β*-GP hydrogel than cells cultured in 100 ng/ml VEGF (Figures 7(f) and 7(g)).

## 4. Discussion

The pulpotomy and direct pulp capping in teeth initially establish a nonbacterial environment and maintain the pulpal vitality for further dentin-pulp complex healing [24]. Under the condition of dental pulp exposure, stem cells in dental pulp provide the potential of pulp self-healing and tertiary dentin formation. Numerous investigations have concerned that the biological behaviors of DPSCs could be affected and amplified by extracellular environment [35]. Nowadays, an increasing focus on the design of new materials has emerged which are capable of driving DPSC migration and differentiation in dental therapy [36]. Among these, the CS/*β*-GP hydrogel has been widely used in drug delivery or tissue engineering systems for its biodegradability, biocompatibility, and antibacterial property [37].

The CS/*β*-GP hydrogel has thermosensitive property. The mixture maintains in the liquid state at room temperature and transforms into gel after 37°C incubation or be injecting into the body [22, 38]. The thermosensitive characteristic has been reported to be helpful in wound healing and bone tissue regeneration [32, 39]. The initial liquid stage can easily flow and fill any target area. Also, the liquid state is useful for encapsulating living cells and therapeutic agents. After the sol-gel transformation in the body, the hydrogel promoted the proliferation of cells. The sol-gel transformation in wound is safe and operable as it does not require externally applied trigger for gelation. Besides, the CS/*β*-GP hydrogels were elevated to be compatible with DPSCs in this and previous studies [39].

Lyophilization resulted in loss of water in the hydrogel; then, the porous structure of dry hydrogel was observed. The porous and hydrous structure allowed DPSC adhesion with embedded cellular synapses in the hydrogels. Numerous investigations indicated that the extracellular microenvironment can have an impact on cell behaviors. The morphology of cells seeded on different carriers showed in different shapes. Studies reported that the odontoblastic cell line was spherical on HA sponge, while flattened with stretching processes on collagen sponge [25]. The difference of cell morphology on these carriers may be related to the adhesion receptor. It was previously demonstrated that odontoblastic cell lines KN-3 adhere to HA through surface markers like CD44 and attach to collagen through the integrins and collagen interaction [40, 41]. In the present study, DPSCs on the CS/*β*-GP hydrogel showed spherical shape. The related adhesion receptors need to be further investigated to identify the adhesion motility of DPSCs on the hydrogel [42, 43].

The AO/EB staining illustrated that the live cells without hydrogel were uniformly distributed on the well, while the live cells grew on the surface of hydrogel showed a status of agminate growth which might be contributed by the advantage of hydrogel for promoting proliferation of DPSCs. The apoptotic cells were stained by EB and less presented on the surface of hydrogels both in the two groups, which was a good proof for the biocompatibility of hydrogel. The well activity of DPSCs was similar with previous studies of human umbilical vein endothelial cells (HUVEC) and mouse embryonic fibroblast cells (NIH 3T3) on other materials composed by chitosan [29, 44]. The results of CCK-8 assay further showed the promoted proliferation of DPSCs with hydrogels. The hydrogel itself is not transparent and may influence the detection of absorbance. DPSCs were cultured in the hydrogel leachates to exclude the hydrogel absorbance in CCK-8 analysis [29]. These evidences were all in agreement with previous studies, suggesting the potential application of CS/*β*-GP hydrogel with great biocompatibility [45].

The previous studies have suggested that preencapsulating drugs in carriers allow a prolonged release of drugs [46], and the sustained delivery capability of CS/*β*-GP hydrogel was also evaluated in consistent with previous findings [47, 48]. The results of ELISA showed the incorporation of VEGF into CS/*β*-GP hydrogel had an initial burst release followed by a sustained release of VEGF over a period of time, and the release cumulation reached a steady level to create a relative steady concentration for cell culture. The similar release status was also observed in CS/*β*-GP hydrogel with other bioactive molecules [19, 22].

As we found the VEGF/CS/*β*-GP hydrogel was able to constantly release VEGF, we further compared the effects on the differentiation of DPSCs between VEGF released from hydrogels and once-added 100 ng/ml VEGF treatment. Generally, agent release from biocompatible materials is related to initial agent loading, agent solubility, carrier material degradation, and so on [49]. In our study, we used the CS/*β*-GP hydrogel that carried 100 ng/ml of VEGF. Compared to the once-added VEGF treatment, DPSCs with VEGF/CS/*β*-GP hydrogel showed more mineralized nodule formation in the late differentiation stage. The higher expression levels of osteogenic/odontogenic markers in the VEGF/CS/*β*-GP hydrogel group were further detected. As a result, we supposed that this delivery system promoted the proliferation and odontogenic differentiation of DPSCs in a period of time, better than 100 ng/ml VEGF treatment without carriers. As described in previous studies [14], VEGF has an effect on odontogenic differentiation of DPSCs, while higher concentrations of VEGF may not always show better effects on DPSCs. Our study yielded similar cell responses to VEGF treatment with different concentrations. Based on the VEGF release behavior from hydrogel, it was suspected that the VEGF concentration in the hydrogel group was lower than that of the 100 ng/ml VEGF group. Besides, the *β*-GP in the CS/*β*-GP hydrogel not only induced the sol-gel transformation at body temperature but also provided organophosphates, as a result, inducing more calcium deposition [50]. All these data suggested that, even though the hydrogel group creates a lower concentration of VEGF in surroundings, the sustained release and steady concentration of VEGF may better contribute to promote the activity and odontogenic differentiation of DPSCs than the initial burst application of VEGF. These effects were consistent with the BMP-2/CS/*β*-GP hydrogel delivery system [22]. On the one hand, the CS/*β*-GP delivery system saves cost and maximizes the effects of VEGF treatment [14, 51, 52]. On the other hand, the delivery system decreased the negative consequence caused by rapid loss of physical stability and bioactivity [22, 32, 44–46]. The transwell technique helps us creating a circumstance to simulate practical application and allowing the VEGF released from hydrogel working on DPSCs.

## 5. Conclusions

In this study, the microstructure and biocompatibility of CS/*β*-GP hydrogel were identified. As a carrier material, the characteristic of sustained releasing VEGF was profiled and contributed to the proliferation and differentiation of DPSCs. Besides, the angiogenesis is another key step in the dental pulp healing. VEGF has been reported to be a potent factor to promote angiogenesis and might be beneficial to form the pulpodentinal complex. However, the advantages of chitosan carrying VEGF on angiogenesis still need further studies. Also, the pharmaceutical applications of hydrogels need further exploration on animal studies and clinical trials [53].

## Figures and Tables

**Table 1:** Primer names and sequences.

Primer names	Primer sequences
*GAPDH*	Forward: GGAGCGAGATCCCTCCAAAAT
	Reverse: GGCTGTTGTCATACTTCTCATGG
*Runx-2*	Forward: CCTTTACTTACACCCCGCCA
	Reverse: GGATCCTGACGAAGTGCCAT
*OCN*	Forward: ATTGTGGCTCACCCTCCATC
	Reverse: CCAGCCTCCAGCACTGTTTA
*OSX*	Forward: TCTGCGGGACTCAACAACTC
	Reverse: TAGCATAGCCTGAGGTGGGT
*ALP*	Forward: CTATCCTGGCTCCGTGCTCC
	Reverse: GTTAACTGATGTTCCAATCCTGCG
*DSPP*	Forward: ATATTGAGGGCTGGAATGGGGA
	Reverse: TTTGTGGCTCCAGCATTGTCA

## Data Availability

The data used to support the findings of the study are available from the corresponding author upon request.
